# Obituary —Dr. Robert Nelson

**Published:** 1868-03

**Authors:** 


### OBITUARY.

At the State Convention of Dentists, held at the city of Utica, December 18th and 19th, 1867, consisting of delegates representing almost every section of the State of New York, for the purpose of considering the subject of some legal enactment to regulate the practice of Dental Surgery, a paper upon the life and death of the late Dr. Robert Nelson, of Albany, was read by A. Westcott, M. D. D., D. S., of Syracuse. After eulogistic remarks from Drs. Foster and Rodgers, of Utica, from Dr. Whitney, of Buffalo, and several others, the accompanying resolutions, on motion of Dr. G. A. Foster, were unanimously adopted by the Convention:

Whereas, The Almighty has seen fit to take from us our beloved friend and brother, Dr. Robert Nelson; and

Whereas, By his honesty of purpose, by his integrity of character, and by the exercise of the most exalted, social, moral, and professional qualities, he has left an example most worthy of our imitation; it is, therefore, by this Convention

*Resolved*, That in this dispensation, the profession of Dental Surgery has sustained a loss which will be felt and deeply mourned by each and all its members.

*Resolved*, That we tender to the family of the deceased our deepest sympathy and condolance, and our prayers that they may be sustained and comforted by Him, who, for His own wise purpose, has called our brother hence.

*Resolved*, That a copy of the paper just read be requested for publication in the different Dental journals; and that it, together with these resolutions, be transmitted to the family of the deceased.

